# A Rare Case of Polymicrogyria in an Elderly Individual With Unique Polygenic Underlining

**DOI:** 10.7759/cureus.74300

**Published:** 2024-11-23

**Authors:** Andrey Frolov, Stuart G Atwood, Miguel A Guzman, John R Martin

**Affiliations:** 1 Department of Surgery - Center for Anatomical Science and Education, Saint Louis University School of Medicine, St. Louis, USA; 2 Department of Pathology, Saint Louis University School of Medicine, St. Louis, USA

**Keywords:** bifrontal, malformation of cortical development, next-generation sequencing, polymicrogyria, whole exome sequencing

## Abstract

Polymicrogyria (PMG) is the most common malformation of cortical development (MCD) and presents as an irregularly patterned cortical surface with numerous small gyri and shallow sulci leading to various neurological deficits including developmental delays, intellectual disability, epilepsy, and language and motor issues. The presentation of PMG varies and is often found in conjunction with other congenital anomalies. Histologically, PMG features an abnormal cortical structure and dyslamination, resulting in its classification as a defect of neuronal migration and organization. Due in part to a variety of etiologies, little is known about the molecular mechanism(s) underlining PMG. To address this gap in knowledge, a case study is presented where an elderly individual with a medical history of unspecified PMG was examined postmortem by using a combination of anatomical, magnetic resonance imaging (MRI), histopathological, and genetic techniques. The results of the study allowed the classification of this case as bifrontal PMG. The genetic screening by whole exome sequencing (WES) on the Illumina Next Generation Sequencing (NGS) platform yielded 83 rare (minor allele frequency, MAF ≤ 0.01) pathological/deleterious variants where none of the respective genes has been previously linked to PMG. However, a subsequent analysis of those variants revealed that a significant number of affected genes were associated with most of the biological processes known to be impaired in PMG thereby pointing toward a polygenic nature in the present case. One of the notable features of the WES dataset was the presence of rare pathological/deleterious variants of genes (*ADGRA2*,* PCDHA1*,* PCDHA12*,* PTK7*,* TPGS1*, and* USP4*) involved in the regulation of Wnt signaling potentially highlighting the latter as an important PMG contributor in the present case. Notably, *ADGRA2* warrants a closer look as a candidate gene for PMG because it not only regulates cortical patterning but has also been recently linked to two cases of bifrontal PMG with multiple congenital anomalies through its compound heterozygous mutations.

## Introduction

Polymicrogyria (PMG) is a malformation of cortical development (MCD) characterized primarily by overfolding of the cortical surface, producing an irregular pattern with numerous small gyri and shallow sulci [1]. With an incidence of 2.3 per 10,000 births [2], PMG is the most common form of MCD, accounting for 20% of all cases [1,3]. PMG has a remarkably heterogeneous phenotype [4] where common clinical presentations may include epilepsy, intellectual disabilities, and deficits in language and/or motor skills. To complicate this matter even further, these symptoms are often observed in conjunction with syndromes bearing multiple congenital anomalies, as well as symptoms specific to the affected cortical areas [1,4]. The phenotypical heterogeneity of PMG could be partially explained by its etiological diversity that in addition to variable genetic underpinnings [4-6] could also include environmental factors such as maternal infection during pregnancy, hypoxia/ischemia, and trauma [5]. Genetic PMG causes are diverse and could include chromosomal aberrations, copy number variations, or mutations involving a single or multiple genes [6]. The most common single-gene mutations target genes encoding cytoskeletal proteins *(TUBA1A, TUBB2B, TUBB3, *and* TUBA8),* genes regulating cell growth *(PIK3R2 *and *FIG4)* and extracellular matrix *(COL18A1 *and *LAMC3)*, as well as neuronal proliferation and migration *(WDR62)* [4,6]. Such remarkable phenotypical and etiological diversity makes the study of addressing molecular mechanism(s) of PMG in humans extremely difficult and could explain the paucity of the available respective information.

Therefore, the main objective of this study was to gain additional insights into the mechanism(s) governing PMG development through a postmortem study with a multifaceted approach including magnetic resonance imaging (MRI), gross anatomical examination and dissection, histopathological examination, as well as genetic screening by the whole exome sequencing (WES) on the next-generation sequencing (NGS) platform. A clearer understanding of the nature of the above pathology may further advance our understanding of the mechanism(s) regulating brain development in humans.

These data were presented in part as an abstract at the Anatomy Connected Meeting on March 24, 2024.

## Case presentation

Anatomical characterization

**Figure 1:**
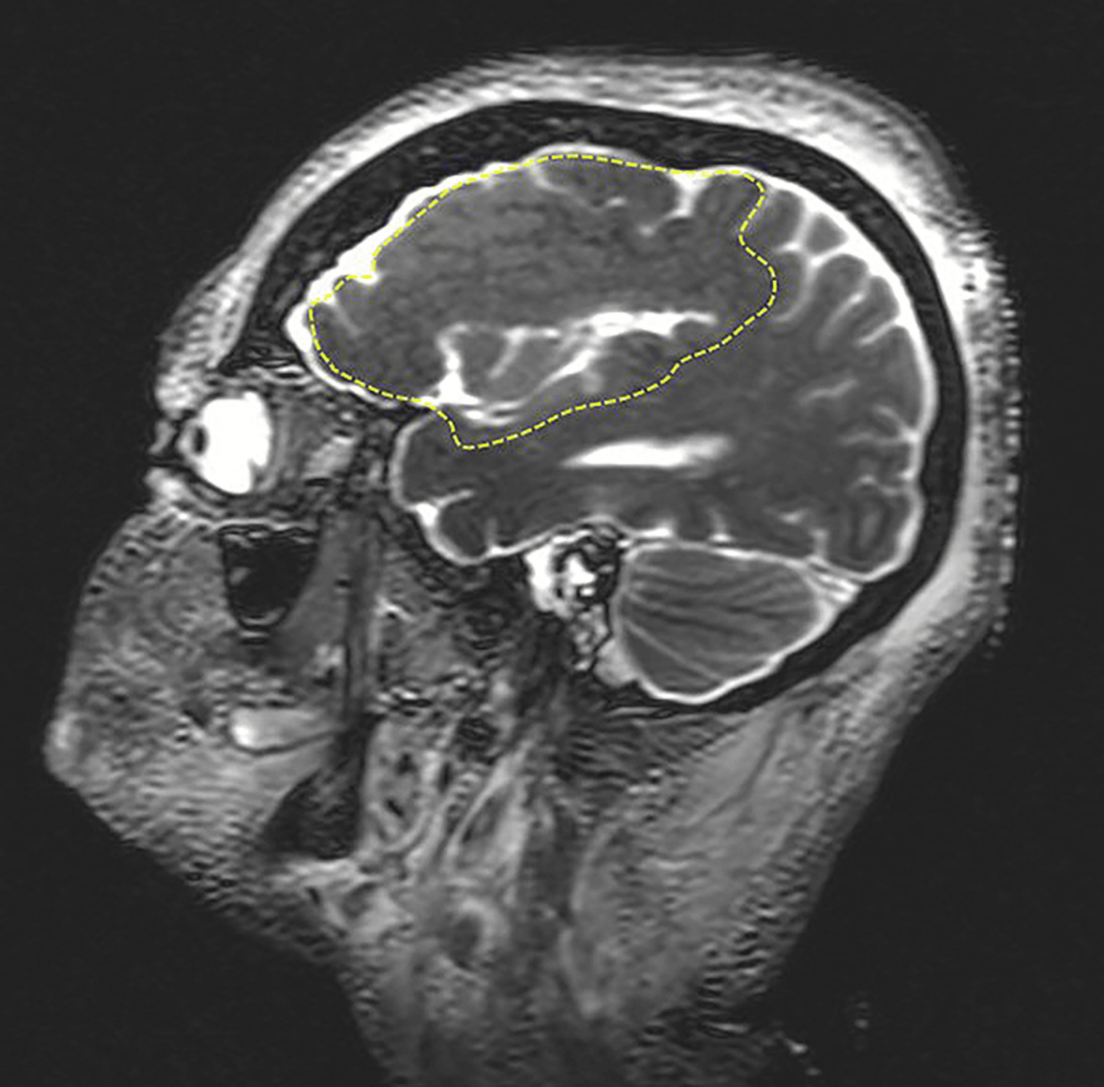
Magnetic resonance imaging of the donor head demonstrating the presence of polymicrogyria (PMG). Parasagittal image of a T2‐weighted MRI is shown. Note the aberrant boundary of the polymicrogyric cortex (depicted by stippled yellow lines) compared to the smooth gray‐white boundary in normal cortical areas.

An 81-year-old female body was received through the Saint Louis University (SLU) Gift Body Program with signed informed consent. Reported medical history included PMG, intellectual disability, static encephalopathy, moderately oral pharyngeal dysphagia, osteoporosis, cerebral palsy, irritable bowel syndrome, deafness, aphasia, ptyalism, and edentia. The cause of death for this individual was acute respiratory failure from acquired pneumonia, leading to hypoxia and severe sepsis. External examination of the donor revealed no external deformities as the extremities were normal with 10 fingers and toes. Body measurements revealed a height of 155 cm or (5’1”) and a head circumference of 50.15 cm. The distinct external characteristic of the donor was a protruding tongue. MRI revealed bifrontal PMG limited to the frontal lobes with small gyri, abnormal gyral patterns, and an irregular gray-white interface (Figure 1). The subcortical areas, brainstem, and cerebellum appeared normally formed with no notable absences or hypoplasia. 

**Figure 2:**
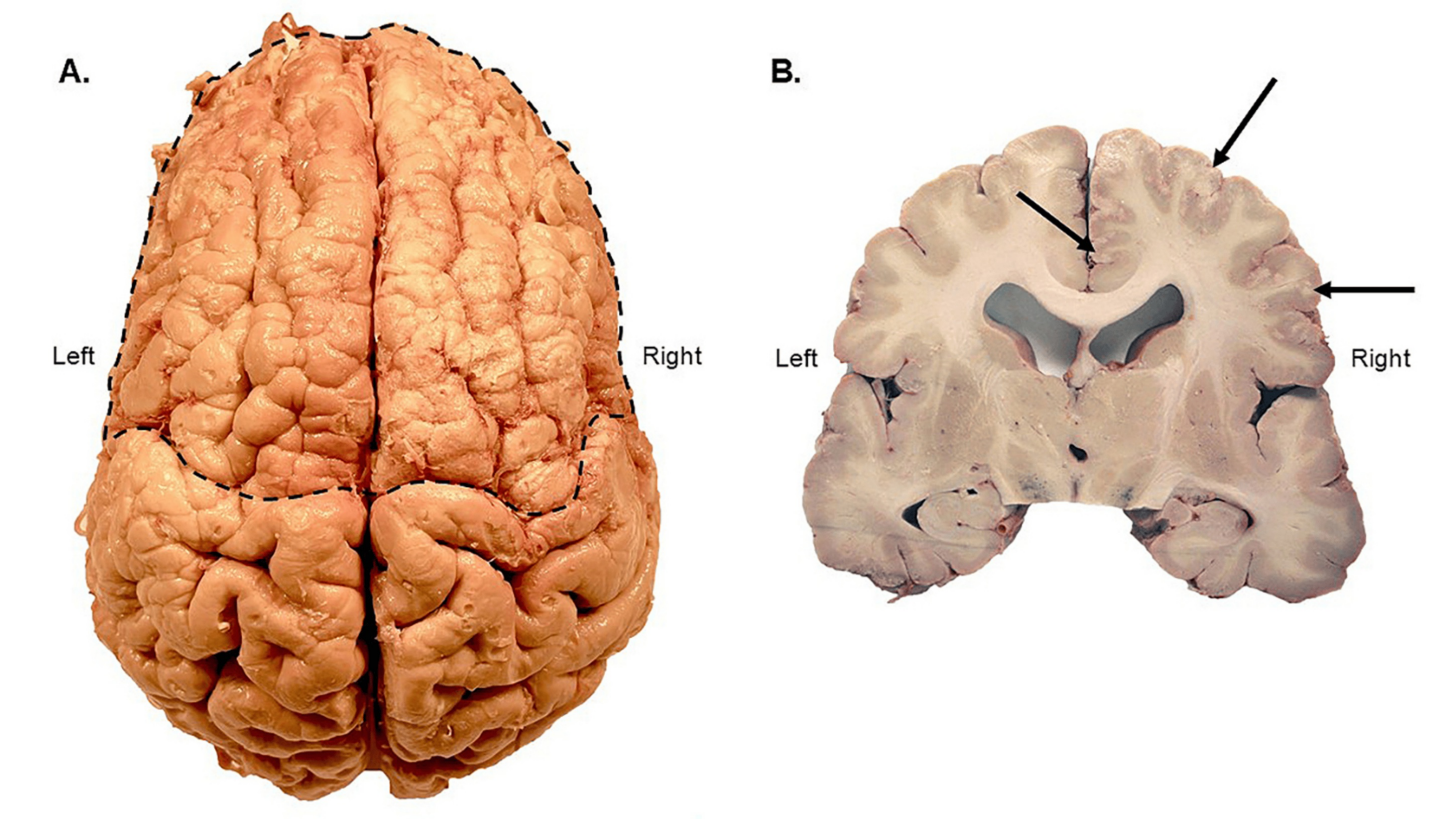
Superior and coronal views of the polymicrogyria (PMG) brain. A. Superior view of the removed brain. Polymicrogyria (PMG) is evident in the outlined area when compared to the typical gyration of the more posterior brain. B. Coronal brain section at the level of the substantia nigra. The intact and fully developed corpus callosum is evident in this section; also of note is the stippled gray matter of the right hemisphere, as indicated by the arrows.  Mild to moderate enlargement of the lateral ventricles was observed.

The gross brain examination confirmed the presence of bifrontal PMG (Figure 2A). Aberrant patterning of the small gyri of the frontal lobes was well defined, in addition to some asymmetrical aberrant patterning in the left parietal and right perisylvian regions, with the apparent absence of the central sulcus of Rolando and precentral gyrus. Coronal sections showed small cortical folds and shallow sulci over the frontal lobes (Figure 2B). Subcortical structures were normally present, and the lateral ventricles showed mild to moderate enlargement, indicative of age-related hydrocephalus ex-vacuo. The cortex of the right frontal lobe showed a stippled grey-white matter boundary in the anterior cingulate gyrus, extending posteriorly in the superior and inferior frontal gyri (Figure 2B).

**Figure 3:**
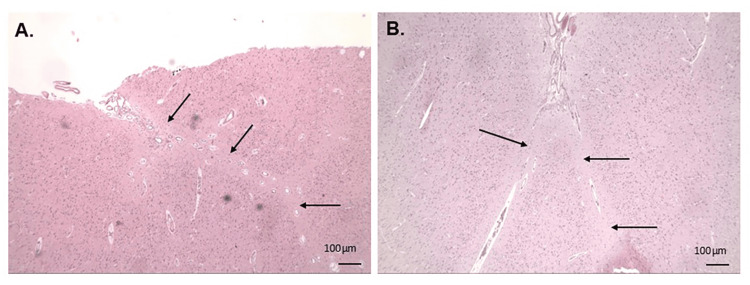
Hematoxylin and eosin (H&E)-stained sections of the right frontal lobe. A. Right cingulate gyrus. The black arrows indicate areas of fused molecular layers between adjacent gyri. Leptomeningeal tissue and intracortical vessels are also evident in the upper part of this image. B. Right middle frontal gyrus. The microscopic image shows the cortical surface with fusion of the molecular layers (black arrows) which appears to extend into the underlying sulcus and includes entrapped leptomeningeal vessels.

Examination of the histological images detailed multiple areas of true gyral fusion between adjacent molecular layers, with evidence of entrapped but otherwise normal, leptomeningeal blood vessels (Figure 3). Additionally, a mild decrease in cortical thickness, with focal neuronal loss in the superficial layers and neuronal dyslamination of the neocortex, was noted. No neuronal heterotopias or dysplastic neurons were noted, and pial surfaces demonstrated no specific abnormalities.

Genetic screening

The post-mortem genetic screening by WES on the Illumina NGS platform and the respective bioinformatics analysis were performed as previously described [7]. The genetic screening revealed rare pathological/deleterious variants in 83 genes (Table 1) with none of the genes listed in the table being previously linked to PMG [6]. Interestingly, among the genes listed in Table 1, there was a group of five pleiotropic genes known to be involved in both neurogenesis and angiogenesis: *ADGRA2* [8,9], *JAG2* [10,11], *LAMA1* [12,13], *SEMA3D* [14,15], and *SYNM* [16]. These data were consistent with a recent hypothesis linking an aberrant hypersprouting angiogenesis to PMG development [17]. To test this hypothesis in the current setting, the histopathological examination of cerebral vasculature was performed but the outcomes were unremarkable (data not shown).

**Table 1:** Complete list of genes with rare pathological/deleterious variants associated with the present case*. * Gene-to-protein name conversion was performed using the GeneCards database; **BBB: blood brain barrier.

Gene	Protein function
A1BG	Alpha-1-B glycoprotein
ABCA3	ATP binding cassette subfamily A member 3; brain development [18]; progenitor cell regulation; neurogenesis [19]; vesicle transport [20]
ABHD5	Abhydrolase domain containing 5, lysophosphatidic acid acyltransferase; skin barrier defect [21]
ACSS1	Acyl-CoA synthetase short chain family member 1
ADGRA2	Adhesion G protein-coupled receptor A2; brain development [8]; cerebral angiogenesis [9]; Wnt signaling [22,23]; bifrontal PMG with multiple congenital anomalies [24]
ADPRHL1	ADP-ribosylhydrolase-like 1 ROCK signaling [25]
BIRC7	Baculoviral IAP repeat containing 7
C19orf57	Break repair meiotic recombinase recruitment factor 1
CAPN12	Calpain 12; angiogenesis [26]; intellectual disability [27]
CARMIL3	Capping protein regulator and myosin 1 linker 3; brain ischemia [28]; cell migration [29]
CCDC198	Coiled-coil domain containing 198. Neurogenesis; progenitor cell regulation; cell migration [30]
CHAF1A	Chromatin assembly factor 1 subunit A. Neurogenesis [31,32]
CHRND	Cholinergic receptor nicotinic delta subunit. Neuromuscular junction formation [33]
CMPK2	Cytidine/uridine monophosphate kinase 2
CYP17A1	Cytochrome P450 family 17 subfamily A member 1; angiogenesis [34]
DENND1B	DENN domain containing 1B; vesicle transport [35]
DHRS2	Dehydrogenase/reductase 2
DNAH3	Dynein axonemal heavy chain 3; motile cilia regulation [36]
FLG2	Filaggrin 2; brain development [37]; skin disease [38]
GCC2	GRIP and coiled-coil domain containing 2
GLYATL3	Glycine-N-acyltransferase like 3
GUSB	Glucuronidase beta
HLA-DRB1	Major histocompatibility complex, class II, DR beta 1
HLA-DRB5	Major histocompatibility complex, class II, DR beta 5
HLTF	Helicase-like transcription factor; neurogenesis/brain development [39]; neuronal cell death [40]
IFI30	IFI30 lysosomal thiol reductase; angiogenesis [41]; neuronal cells death [42]
JAG2	Jagged canonical notch ligand 2; progenitor cell regulation [11]; angiogenesis [10]
KMT2C	Lysine methyltransferase 2C; neurogenesis/brain development [43,44]
KRT19	Keratin 19
KRT86	Keratin 86
KRTAP3-2	Keratin associated protein 3-2
LAMA1	Laminin subunit alpha 1. Neurogenesis/brain development [12]; angiogenesis [13]
LONP1	Lon Peptidase 1, mitochondrial. Mitochondrial encephalopathy [45]; neuronal cell death [46]
LRGUK	Leucine-rich repeats and guanylate kinase domain containing. Motile cilia regulation [47]
LRRC58	Leucine-rich repeat containing 58. Neuroinflammation [48,49]
MDH1	Malate dehydrogenase 1. Energy metabolism [50]; neuronal cell death [51]
MGST2	Microsomal glutathione S-transferase 2. Neuronal cell death [52]; angiogenesis [53]
MLXIP	MLX interacting protein. Brain development/neurogenesis; progenitor cell regulation; energy metabolism [54]
MPEG1	Macrophage expressed 1
MRPL57	Mitochondrial ribosomal protein L57. Energy metabolism [55]
MS4A15	Membrane spanning 4-domains A15. Brain development/neurogenesis [56]
MTUS1	Microtubule-associated scaffold protein 1. Rac signaling [57]
MYO7B	Myosin VIIB. Vesicle transport [58]. Possibly linked to brain development/neurogenesis [59]
NGLY1	N-glycanase 1. Brain development/neurogenesis [60,61]; neuronal cell death [62]
NMU	Neuromedin U
OBSCN	Obscurin, cytoskeletal calmodulin, and titin-interacting. Brain development/neurogenesis [63]
OPN3	Opsin 3
OR2S2	Olfactory receptor family 2 subfamily S member 2. BBB** regulation [64]
PAH	Phenylalanine hydroxylase
PCDHA1	Protocadherin alpha 1. Brain development/neurogenesis [65,66]; Wnt signaling [67]
PCDHA12	Protocadherin alpha 12. Brain development/neurogenesis [68]; Wnt signaling [69,70]
PGK2	Phosphoglycerate kinase 2
PHKB	Phosphorylase kinase regulatory subunit beta
PI15	Peptidase inhibitor 15
PLEKHG3	Pleckstrin homology and RhoGEF domain containing G3. Brain development/neurogenesis [71,72]; Rho signaling [72]; linked to mild intellectual disability [73]
PSG6	Pregnancy-specific beta-1-glycoprotein 6. Brain development/neurogenesis [74]
PTK7	Protein tyrosine kinase 7 (Inactive). Brain development/neurogenesis [75,76]; Wnt signaling [75,77]; cell migration [77]
PTPRN2	Protein tyrosine phosphatase receptor type N2. TGFβ signaling [78]; cerebral vasculature remodeling [79]
RGR	Retinal G protein coupled receptor
RIT2	Ras like without CAAX 2. Brain development/neurogenesis; Ras signaling [80]
RSL1D1	Ribosomal L1 domain containing 1 (cellular senescence-inhibited gene protein, CSIG). Cell proliferation and senescence [81]; linked to child cognitive development [82]
SEMA3D	Semaphorin 3D. Signaling [83]; linked to cognitive impairment [14]; angiogenesis [15]
SKIV2L	SKI2 subunit of superkiller complex
SLC26A1	Solute carrier family 26 member 1
SLC9C1	Solute carrier family 9 member C1. Sperm-specific component of motile cilia [84]
SYNM	Synemin. Neurogenesis; progenitor cell regulation; cell migration [16]
TIAM2	TIAM Rac1 associated GEF 2. Brain development/neurogenesis; Rho signaling [85]
TLN2	Talin 2
TMEM43	Transmembrane protein 43
TPGS1	Tubulin polyglutamylase complex Subunit 1. Cilia regulation [86]
TTN	Titin
TUT1	Terminal uridylyl transferase 1, U6 SnRNA-specific. Brain development/neurogenesis [87]; neuronal cell death [88]
TYK2	Tyrosine kinase 2. Brain development/neurogenesis [89]. Jak/Stat signaling; neuronal cell death [90]
UBE4B	Ubiquitination factor E4B. Brain development/neurogenesis; progenitor cell regulation; mTOR signaling [91]; neuronal cell death [92]
UGT1A7	UDP glucuronosyltransferase family 1 member A7; BBB regulation [93]
UNKL	Unk like zinc finger. Neurogenesis [94]
UPK1A	Uroplakin 1A
USP4	Ubiquitin specific peptidase 4. Brain development/neurogenesis [95,96]; neuronal cell death [97]; Wnt signaling [98,99]
ZBED9	SCAN domain containing 3 (SCAND3). Potential biomarker for mild cognitive impairment [100]
ZC3H3	Zinc finger CCCH-type containing 3
ZNF227	Zinc finger protein 227
ZNF540	Zinc finger protein 540
ZYX	Zyxin. Brain development/neurogenesis [101]; tight junction/BBB regulation [102]; Shh signaling [103]; cell migration [104]

## Discussion

The current report provides additional insights into the molecular mechanism(s) underlining PMG development. The examination of the individual’s brain by anatomical, MRI, and histological techniques allowed the classification of the observed MCD as bifrontal PMG with an absence of visible musculoskeletal defects often associated with PMG [1]. The performed genetic analysis provided several important insights into its development.

First, there was a plethora of genes linked to the biological processes that could be perturbed in PMG (Table 1) [1,105] thereby being consistent with the polygenic underlining of the present case. *Second,* there were several genes involved in the regulation of cilia function including both of its types, primary and motile (Table 1). Given the crucial role of motile cilia in neurodevelopment through regulation of cerebrospinal fluid (CSF) fluid flow [106,107] and ventricular development [108,109], as well as the absence of non-age related hydrocephalus pathology in the donor’s brain (Figure 2), one may conclude that PMG in the present case was not mediated by motile cilia but was rather associated with an input from the aberrant primary cilia-mediated signaling [110,111]. Such signaling aberration could be explained, at least in part, by the abnormal primary cilia formation driven by the mutated *TPGS1* (Table 1) [86] and by impaired Wnt signaling, due to mutations in *ADGRA2, PCDHA1, PCDHA12, PTK7,* and *USP4* (Table 1), which use primary cilia as a signaling platform [112].

Third, *ADGRA2,* also known as *GPR124,* was a very interesting gene because it not only positively regulates canonical Wnt signaling by increasing Wnt7 availability for Frizzled [22, 23], but by virtue of its compound heterozygous mutations, it has also been recently linked to two bifrontal PMG cases with multiple congenital anomalies [24]. It should also be noted that the other member of the same gene family, *ADGRG1 (GPR56)* with an autosomal recessive variant, was reported to be associated with bilateral frontoparietal PMG [113, 114]. Therefore, the results of the current report and the data presented in [24] merit a closer look at *AGDRA2* as a potentially causative gene in bifrontal PMG.

Fourth, the other notable feature of the genetic screening dataset was the presence of the biallelic *FLG2* variant (NM_001014342:exon3:c.C2606T:p.S869F; MAF = 4.21x10-6). *FLG2* is known for its association with skin diseases including atopic dermatitis [38], as well as for its link to neurodevelopmental aberrations, leading to autism spectrum disorder (ASD) with a prenatal excessive cortical expansion frequently seen in ASD children [37]. Such a link between neuro- and ectodermal development supports a hypothesis regarding the existence of the skin-brain axis, which could interdependently regulate both processes [37]. Unfortunately, due to the condition of cadaveric tissue subjected to the embalming solution, it was impossible to correctly assess the putative epidermal pathology in the donor and, therefore, evaluate the involvement of the skin-brain axis in the current case of bifrontal PMG. However, probing similar PMG cases for the autosomal recessive *FLG2* mutations and atopic syndromes antemortem would be worth pursuing.

## Conclusions

The current rare case of bifrontal PMG in an elderly individual provided a unique opportunity to gain additional insights into the molecular mechanism(s) of PMG. Our results highlight the polygenic nature of PMG and the potential involvement of impaired Wnt signaling with the involvement of primary cilia deregulation and a direct Wnt signaling disruption. Our results also warrant additional studies on the *ADGRA2* gene as well as probing the skin-brain axis for their participation in the development of the cerebral cortex in humans.

## References

[1] Stutterd CA, Leventer RJ (2014). Polymicrogyria: a common and heterogeneous malformation of cortical development. Am J Med Genet C Semin Med Genet.

[2] Kolbjer S, Martín Muñoz DA, Örtqvist AK, Pettersson M, Hammarsjö A, Anderlid BM, Dahlin M (2023). Polymicrogyria: epidemiology, imaging, and clinical aspects in a population-based cohort. Brain Commun.

[3] Leventer RJ, Phelan EM, Coleman LT, Kean MJ, Jackson GD, Harvey AS (1999). Clinical and imaging features of cortical malformations in childhood. Neurology.

[4] Squier W, Jansen A (2014). Polymicrogyria: pathology, fetal origins and mechanisms. Acta Neuropathol Commun.

[5] Jansen A, Andermann E (2005). Genetics of the polymicrogyria syndromes. J Med Genet.

[6] James J, Iype M, Surendran MO, Anitha A, Thomas SV (2022). The genetic landscape of polymicrogyria. Ann Indian Acad Neurol.

[7] Frolov A, Guzman MA, Hayat G, Martin JR 3rd (2024). Two cases of sporadic amyotrophic lateral sclerosis with contrasting clinical phenotypes: genetic insights. Cureus.

[8] Sreepada A, Tiwari M, Pal K (2022). Adhesion G protein-coupled receptor gluing action guides tissue development and disease. J Mol Med (Berl).

[9] Bostaille N, Gauquier A, Stainier DY, Raible DW, Vanhollebeke B (2017). Defective adgra2 (gpr124) splicing and function in zebrafish ouchless mutants. Development.

[10] Yao Y, Yao J, Radparvar M, Blazquez-Medela AM, Guihard PJ, Jumabay M, Boström KI (2013). Reducing Jagged 1 and 2 levels prevents cerebral arteriovenous malformations in matrix Gla protein deficiency. Proc Natl Acad Sci U S A.

[11] Rabadán MA, Cayuso J, Le Dréau G (2012). Jagged2 controls the generation of motor neuron and oligodendrocyte progenitors in the ventral spinal cord. Cell Death Differ.

[12] Ichikawa-Tomikawa N, Ogawa J, Douet V (2012). Laminin α1 is essential for mouse cerebellar development. Matrix Biol.

[13] Edwards MM, McLeod DS, Grebe R, Heng C, Lefebvre O, Lutty GA (2011). Lama1 mutations lead to vitreoretinal blood vessel formation, persistence of fetal vasculature, and epiretinal membrane formation in mice. BMC Dev Biol.

[14] Chen CY, Chao YM, Cho CC (2023). Cerebral Semaphorin3D is a novel risk factor for age-associated cognitive impairment. Cell Commun Signal.

[15] Degenhardt K, Singh MK, Aghajanian H (2013). Semaphorin 3d signaling defects are associated with anomalous pulmonary venous connections. Nat Med.

[16] Izmiryan A, Franco CA, Paulin D, Li Z, Xue Z (2009). Synemin isoforms during mouse development: multiplicity of partners in vascular and neuronal systems. Exp Cell Res.

[17] Klostranec JM, Chen L, Mathur S, McDonald J, Faughnan ME, Ratjen F, Krings T (2019). A theory for polymicrogyria and brain arteriovenous malformations in HHT. Neurology.

[18] Tachikawa M, Watanabe M, Hori S, Fukaya M, Ohtsuki S, Asashima T, Terasaki T (2005). Distinct spatio-temporal expression of ABCA and ABCG transporters in the developing and adult mouse brain. J Neurochem.

[19] Lin T, Islam O, Heese K (2006). ABC transporters, neural stem cells and neurogenesis--a different perspective. Cell Res.

[20] García-Sanz P, M F G Aerts J, Moratalla R (2021). The role of cholesterol in α-synuclein and Lewy body pathology in GBA1 Parkinson's disease. Mov Disord.

[21] Lord CC, Thomas G, Brown JM (2013). Mammalian alpha beta hydrolase domain (ABHD) proteins: lipid metabolizing enzymes at the interface of cell signaling and energy metabolism. Biochim Biophys Acta.

[22] Vallon M, Yuki K, Nguyen TD (2018). A RECK-WNT7 receptor-ligand interaction enables isoform-specific regulation of Wnt bioavailability. Cell Rep.

[23] Eubelen M, Bostaille N, Cabochette P (2018). A molecular mechanism for Wnt ligand-specific signaling. Science.

[24] Chong K, Keunen J, Staines A (2024). The ADGRA2 gene is associated with multiple fetal brain anomalies in humans. Genet Med Open.

[25] Tian L, Guo T, Wu F (2023). The pseudoenzyme ADPRHL1 affects cardiac function by regulating the ROCK pathway. Stem Cell Res Ther.

[26] Veluchamy A, Ballerini L, Vitart V (2019). Novel genetic locus influencing retinal venular tortuosity is also associated with risk of coronary artery disease. Arterioscler Thromb Vasc Biol.

[27] Riazuddin S, Hussain M, Razzaq A (2017). Exome sequencing of Pakistani consanguineous families identifies 30 novel candidate genes for recessive intellectual disability. Mol Psychiatry.

[28] Yeh SJ, Hsu PH, Yeh TY (2021). Capping protein regulator and myosin 1 linker 3 (CARMIL3) as a molecular signature of ischemic neurons in the DWI-T2 mismatch areas after stroke. Front Mol Neurosci.

[29] Stark BC, Gao Y, Sepich DS (2022). CARMIL3 is important for cell migration and morphogenesis during early development in zebrafish. Dev Biol.

[30] Niklasson CU, Fredlund E, Monni E (2021). Hypoxia inducible factor-2α importance for migration, proliferation, and self-renewal of trunk neural crest cells. Dev Dyn.

[31] Tao L, Moreno-Smith M, Ibarra-García-Padilla R (2021). CHAF1A blocks neuronal differentiation and promotes neuroblastoma oncogenesis via metabolic reprogramming. Adv Sci (Weinh).

[32] Gascón S, Masserdotti G, Russo GL, Götz M (2017). Direct neuronal reprogramming: achievements, hurdles, and new roads to success. Cell Stem Cell.

[33] Ohkawara B, Kurokawa M, Kanai A (2024). Transcriptome profile of subsynaptic myonuclei at the neuromuscular junction in embryogenesis. J Neurochem.

[34] Song Y, Hu R, Li F (2024). In view of ovarian steroidogenesis and luteal construction to explore the effects of Bushen Huoxue recipe in mice of ovarian hyperstimulation. J Ethnopharmacol.

[35] Marat AL, McPherson PS (2010). The connecdenn family, Rab35 guanine nucleotide exchange factors interfacing with the clathrin machinery. J Biol Chem.

[36] De Ita M, Gaytán-Cervantes J, Cisneros B (2022). Clustering of genetic anomalies of cilia outer dynein arm and central apparatus in patients with transposition of the great arteries. Genes (Basel).

[37] Jameson C, Boulton KA, Silove N, Nanan R, Guastella AJ (2023). Ectodermal origins of the skin-brain axis: a novel model for the developing brain, inflammation, and neurodevelopmental conditions. Mol Psychiatry.

[38] Berna R, Mitra N, Hoffstad O, Wubbenhorst B, Nathanson KL, Margolis DJ (2022). Uncommon variants in FLG2 and TCHHL1 are associated with remission of atopic dermatitis in a large longitudinal US cohort. Arch Dermatol Res.

[39] Elserafy M, Abugable AA, Atteya R, El-Khamisy SF (2018). Rad5, HLTF, and SHPRH: a fresh view of an old story. Trends Genet.

[40] Helmer RA, Foreman O, Dertien JS, Panchoo M, Bhakta SM, Chilton BS (2013). Role of helicase-like transcription factor (hltf) in the G2/m transition and apoptosis in brain. PLoS One.

[41] Wang X, Ge X, Qin Y, Liu D, Chen C (2022). Ifi30 is required for sprouting angiogenesis during caudal vein plexus formation in zebrafish. Front Physiol.

[42] Satoh J, Obayashi S, Misawa T, Tabunoki H, Yamamura T, Arima K, Konno H (2008). Neuromyelitis optica/Devic's disease: gene expression profiling of brain lesions. Neuropathology.

[43] Shen E, Shulha H, Weng Z, Akbarian S (2014). Regulation of histone H3K4 methylation in brain development and disease. Philos Trans R Soc Lond B Biol Sci.

[44] Vallianatos CN, Iwase S (2015). Disrupted intricacy of histone H3K4 methylation in neurodevelopmental disorders. Epigenomics.

[45] Besse A, Brezavar D, Hanson J, Larson A, Bonnen PE (2020). LONP1 de novo dominant mutation causes mitochondrial encephalopathy with loss of LONP1 chaperone activity and excessive LONP1 proteolytic activity. Mitochondrion.

[46] Nimmo GA, Venkatesh S, Pandey AK (2019). Bi-allelic mutations of LONP1 encoding the mitochondrial LonP1 protease cause pyruvate dehydrogenase deficiency and profound neurodegeneration with progressive cerebellar atrophy. Hum Mol Genet.

[47] Samsel Z, Sekretarska J, Osinka A, Wloga D, Joachimiak E (2021). Central apparatus, the molecular kickstarter of ciliary and flagellar nanomachines. Int J Mol Sci.

[48] Luan W, Li M, Wu C, Shen X, Sun Z (2022). Proteomic dissimilarities of primary microglia and BV2 cells under stimuli. Eur J Neurosci.

[49] Sousa C, Golebiewska A, Poovathingal SK (2018). Single-cell transcriptomics reveals distinct inflammation-induced microglia signatures. EMBO Rep.

[50] Guo X, Park JE, Gallart-Palau X, Sze SK (2020). Oxidative damage to the TCA cycle enzyme MDH1 dysregulates bioenergetic enzymatic activity in the aged murine brain. J Proteome Res.

[51] Wang M, Zhou C, Yu L (2022). Upregulation of MDH1 acetylation by HDAC6 inhibition protects against oxidative stress-derived neuronal apoptosis following intracerebral hemorrhage. Cell Mol Life Sci.

[52] Slotkin TA, Seidler FJ (2007). Comparative developmental neurotoxicity of organophosphates in vivo: transcriptional responses of pathways for brain cell development, cell signaling, cytotoxicity and neurotransmitter systems. Brain Res Bull.

[53] Li J, Bruns AF, Hou B (2015). Orai3 surface accumulation and calcium entry evoked by vascular endothelial growth factor. Arterioscler Thromb Vasc Biol.

[54] de Toledo VH, Feltrin AS, Barbosa AR, Tahira AC, Brentani H (2022). Sex differences in gene regulatory networks during mid-gestational brain development. Front Hum Neurosci.

[55] Barshad G, Blumberg A, Cohen T, Mishmar D (2018). Human primitive brain displays negative mitochondrial-nuclear expression correlation of respiratory genes. Genome Res.

[56] Paban V, Loriod B, Villard C (2017). Omics analysis of mouse brain models of human diseases. Gene.

[57] Bai X, Zhou Y, Ouyang N, Liu L, Huang X, Tian J, Lv T (2019). A de novo Mutation in the MTUS1 Gene Decreases the Risk of Non-compaction of Ventricular Myocardium via the Rac1/Cdc42 Pathway. Front Pediatr.

[58] Zhang Q, Xu Y, Lee J (2020). A myosin-7B-dependent endocytosis pathway mediates cellular entry of α-synuclein fibrils and polycation-bearing cargos. Proc Natl Acad Sci U S A.

[59] Perrone E, Perez AB, D'Almeida V (2021). Clinical and molecular evaluation of 13 Brazilian patients with Gomez-López-Hernández syndrome. Am J Med Genet A.

[60] Asahina M, Fujinawa R, Hirayama H, Tozawa R, Kajii Y, Suzuki T (2021). Reversibility of motor dysfunction in the rat model of NGLY1 deficiency. Mol Brain.

[61] Lin VJ, Hu J, Zolekar A (2022). Deficiency of N-glycanase 1 perturbs neurogenesis and cerebral development modeled by human organoids. Cell Death Dis.

[62] Asahina M, Fujinawa R, Nakamura S, Yokoyama K, Tozawa R, Suzuki T (2020). Ngly1 -/- rats develop neurodegenerative phenotypes and pathological abnormalities in their peripheral and central nervous systems. Hum Mol Genet.

[63] Raeker MO, Bieniek AN, Ryan AS, Tsai HJ, Zahn KM, Russell MW (2010). Targeted deletion of the zebrafish obscurin A RhoGEF domain affects heart, skeletal muscle and brain development. Dev Biol.

[64] Santos CR, Duarte AC, Costa AR, Tomás J, Quintela T, Gonçalves I (2019). The senses of the choroid plexus. Prog Neurobiol.

[65] Bruno LP, Doddato G, Valentino F (2021). New candidates for autism/intellectual disability identified by whole-exome sequencing. Int J Mol Sci.

[66] Sjöstedt E, Fagerberg L, Hallström BM (2015). Defining the human brain proteome using transcriptomics and antibody-based profiling with a focus on the cerebral cortex. PLoS One.

[67] Asad M, Wong MK, Tan TZ (2014). FZD7 drives in vitro aggressiveness in Stem-A subtype of ovarian cancer via regulation of non-canonical Wnt/PCP pathway. Cell Death Dis.

[68] Stathopoulos S, Gaujoux R, Lindeque Z, Mahony C, Van Der Colff R, Van Der Westhuizen F, O'Ryan C (2020). DNA methylation associated with mitochondrial dysfunction in a South African autism spectrum disorder cohort. Autism Res.

[69] Zhang J, Cao H, Zhang B (2013). Berberine potently attenuates intestinal polyps growth in ApcMin mice and familial adenomatous polyposis patients through inhibition of Wnt signalling. J Cell Mol Med.

[70] Hemnes AR, Zhao M, West J (2016). Critical genomic networks and vasoreactive variants in idiopathic pulmonary arterial hypertension. Am J Respir Crit Care Med.

[71] Griswold AJ, Ma D, Sacharow SJ (2011). A de novo 1.5 Mb microdeletion on chromosome 14q23.2-23.3 in a patient with autism and spherocytosis. Autism Res.

[72] Kouchi Z, Kojima M (2022). Function of SYDE C2-RhoGAP family as signaling hubs for neuronal development deduced by computational analysis. Sci Rep.

[73] Lybaek H, Øyen N, Fauske L, Houge G (2008). A 2.1 Mb deletion adjacent but distal to a 14q21q23 paracentric inversion in a family with spherocytosis and severe learning difficulties. Clin Genet.

[74] Gibitova EA, Dobrynin PV, Pomerantseva EA (2022). A study of the genomic variations associated with autistic spectrum disorders in a Russian cohort of patients using whole-exome sequencing. Genes (Basel).

[75] Shnitsar I, Borchers A (2008). PTK7 recruits dsh to regulate neural crest migration. Development.

[76] Paudyal A, Damrau C, Patterson VL (2010). The novel mouse mutant, chuzhoi, has disruption of Ptk7 protein and exhibits defects in neural tube, heart and lung development and abnormal planar cell polarity in the ear. BMC Dev Biol.

[77] Berger H, Wodarz A, Borchers A (2017). PTK7 faces the Wnt in development and disease. Front Cell Dev Biol.

[78] Lee SA, Mefford JA, Huang Y (2016). Host genetic predictors of the kynurenine pathway of tryptophan catabolism among treated HIV-infected Ugandans. AIDS.

[79] Haqqani AS, Mianoor Z, Star AT (2023). Proteome profiling of brain vessels in a mouse model of cerebrovascular pathology. Biology (Basel).

[80] Daneshmandpour Y, Darvish H, Emamalizadeh B (2018). RIT2: responsible and susceptible gene for neurological and psychiatric disorders. Mol Genet Genomics.

[81] Ma L, Zhao W, Zheng Q, Chen T, Qi J, Li G, Tong T (2016). Ribosomal L1 domain and lysine-rich region are essential for CSIG/ RSL1D1 to regulate proliferation and senescence. Biochem Biophys Res Commun.

[82] de Mendonça Filho EJ, Barth B, Bandeira DR (2021). Cognitive development and brain gray matter susceptibility to prenatal adversities: moderation by the prefrontal cortex brain-derived neurotrophic factor gene co-expression network. Front Neurosci.

[83] Aghajanian H, Choi C, Ho VC, Gupta M, Singh MK, Epstein JA (2014). Semaphorin 3d and semaphorin 3e direct endothelial motility through distinct molecular signaling pathways. J Biol Chem.

[84] Vyklicka L, Lishko PV (2020). Dissecting the signaling pathways involved in the function of sperm flagellum. Curr Opin Cell Biol.

[85] Chiu CY, Leng S, Martin KA, Kim E, Gorman S, Duhl DM (1999). Cloning and characterization of T-cell lymphoma invasion and metastasis 2 (TIAM2), a novel guanine nucleotide exchange factor related to TIAM1. Genomics.

[86] Wang L, Paudyal SC, Kang Y (2022). Regulators of tubulin polyglutamylation control nuclear shape and cilium disassembly by balancing microtubule and actin assembly. Cell Res.

[87] Karaca E, Harel T, Pehlivan D (2015). Genes that affect brain structure and function identified by rare variant analyses of mendelian neurologic disease. Neuron.

[88] Kim J, Nakamura J, Hamada C (2020). USP15 deubiquitinates TUT1 associated with RNA metabolism and maintains cerebellar homeostasis. Mol Cell Biol.

[89] Chung BM, Kang HC, Han SY (2006). Jak2 and Tyk2 are necessary for lineage-specific differentiation, but not for the maintenance of self-renewal of mouse embryonic stem cells. Biochem Biophys Res Commun.

[90] Wan J, Fu AK, Ip FC (2010). Tyk2/STAT3 signaling mediates beta-amyloid-induced neuronal cell death: implications in Alzheimer's disease. J Neurosci.

[91] Kong X, Shu X, Wang J (2022). Fine-tuning of mTOR signaling by the UBE4B-KLHL22 E3 ubiquitin ligase cascade in brain development. Development.

[92] Zeinab RA, Wu H, Sergi C, Leng R (2012). UBE4B: a promising regulatory molecule in neuronal death and survival. Int J Mol Sci.

[93] Ouzzine M, Gulberti S, Ramalanjaona N, Magdalou J, Fournel-Gigleux S (2014). The UDP-glucuronosyltransferases of the blood-brain barrier: their role in drug metabolism and detoxication. Front Cell Neurosci.

[94] Murn J, Zarnack K, Yang YJ (2015). Control of a neuronal morphology program by an RNA-binding zinc finger protein, Unkempt. Genes Dev.

[95] Zhao Q, Li Y, Du X, Chen X, Jiao Q, Jiang H (2021). Effects of deubiquitylases on the biological behaviors of neural stem cells. Dev Neurobiol.

[96] Bouron A, Fauvarque MO (2022). Genome-wide analysis of genes encoding core components of the ubiquitin system during cerebral cortex development. Mol Brain.

[97] Liu C, Liu C, Liu H (2017). Increased expression of ubiquitin-specific protease 4 participates in neuronal apoptosis after intracerebral hemorrhage in adult rats. Cell Mol Neurobiol.

[98] Zhao B, Schlesiger C, Masucci MG, Lindsten K (2009). The ubiquitin specific protease 4 (USP4) is a new player in the Wnt signalling pathway. J Cell Mol Med.

[99] Yun SI, Kim HH, Yoon JH (2015). Ubiquitin specific protease 4 positively regulates the WNT/β-catenin signaling in colorectal cancer. Mol Oncol.

[100] Li QS, Vasanthakumar A, Davis JW, Idler KB, Nho K, Waring JF, Saykin AJ (2021). Association of peripheral blood DNA methylation level with Alzheimer's disease progression. Clin Epigenetics.

[101] Martynova NIu, Ermolina LV, Eroshkin FM, Gioeva FK, Zaraĭskiĭ AG (2008). Transcriptional factor Xanf1 interacts with the focal adhesion protein zyxin in the early development of the Xenopus laevis brain. Bioorg Khim.

[102] Miyazaki Y, Yusa T, Matsuo S, Terauchi Y, Miyazaki S (2014). Zyxin modulates the transmigration of Haemophilus influenzae to the central nervous system. Virulence.

[103] Martynova NY, Ermolina LV, Ermakova GV, Eroshkin FM, Gyoeva FK, Baturina NS, Zaraisky AG (2013). The cytoskeletal protein Zyxin inhibits Shh signaling during the CNS patterning in Xenopus laevis through interaction with the transcription factor Gli1. Dev Biol.

[104] Kang X, Deng Y, Cao Y, Huo Y, Luo J (2021). Zyxin mediates vascular repair via endothelial migration promoted by forskolin in mice. Front Physiol.

[105] Llinares-Benadero C, Borrell V (2019). Deconstructing cortical folding: genetic, cellular and mechanical determinants. Nat Rev Neurosci.

[106] Ringers C, Olstad EW, Jurisch-Yaksi N (2020). The role of motile cilia in the development and physiology of the nervous system. Philos Trans R Soc Lond B Biol Sci.

[107] Kumar V, Umair Z, Kumar S, Goutam RS, Park S, Kim J (2021). The regulatory roles of motile cilia in CSF circulation and hydrocephalus. Fluids Barriers CNS.

[108] Olstad EW, Ringers C, Hansen JN (2019). Ciliary beating compartmentalizes cerebrospinal fluid flow in the brain and regulates ventricular development. Curr Biol.

[109] D'Gama PP, Qiu T, Cosacak MI (2021). Diversity and function of motile ciliated cell types within ependymal lineages of the zebrafish brain. Cell Rep.

[110] Kheradmand Kia S, Verbeek E, Engelen E (2012). RTTN mutations link primary cilia function to organization of the human cerebral cortex. Am J Hum Genet.

[111] Guemez-Gamboa A, Coufal NG, Gleeson JG (2014). Primary cilia in the developing and mature brain. Neuron.

[112] Niehrs C, Da Silva F, Seidl C (2024). Cilia as Wnt signaling organelles. Trends Cell Biol.

[113] Piao X, Hill RS, Bodell A (2004). G protein-coupled receptor-dependent development of human frontal cortex. Science.

[114] Piao X, Chang BS, Bodell A (2005). Genotype-phenotype analysis of human frontoparietal polymicrogyria syndromes. Ann Neurol.

